# Machine Learning‐Based High‐Throughput Screening, Molecular Modeling and Quantum Chemical Analysis to Investigate *Mycobacterium tuberculosis* MetRS Inhibitors

**DOI:** 10.1002/open.202400460

**Published:** 2025-02-25

**Authors:** Rajesh Maharjan, Kalpana Gyawali, Arjun Acharya, Madan Khanal, Kamal Khanal, Mohan Bahadur Kshetri, Madhav Prasad Ghimire, Tika Ram Lamichhane

**Affiliations:** ^1^ Central Department of Physics Tribhuvan University Kathmandu 44600 Nepal

**Keywords:** machine learning, *mycobacterium tuberculosis*, molecular docking, molecular dynamics simulations, binding free energy

## Abstract

The increasing drug resistance of *Mycobacterium tuberculosis (Mtb)* complicates its effective treatment and often leads to severe side effects. This research aims to pinpoint the potential drug candidates targeting *Mtb* methionyl‐tRNA synthetase (*Mtb*MetRS) using *in silico* techniques. Employing machine learning algorithms, including Random Forest, Extra Trees, and Nu‐Support Vector, a voting classifier was built to screen 10 million molecules. A total of 590 molecules were filtered and analyzed for mutagenicity and other toxicities, resulting in 169 candidates for molecular docking. Among these, 1‐[4‐(1,3‐benzodioxol‐5‐ylmethyl)piperazin‐1‐yl]‐2‐phenylsulfanylethanone (L1) and 1‐ethyl‐6‐fluoro‐4‐oxo‐7‐(4‐pentanoylpiperazin‐1‐yl)quinoline‐3‐carboxylic acid (L2) demonstrated strong binding affinities (−12.74 kcal/mol for L1 and −11.83 kcal/mol for L2) and favorable pharmacokinetic properties. MM/PBSA, DFT calculations, and LD_50_ values supported their stability, reactive nature, and safer toxicity profile, respectively. L1 and L2 are investigated as potential inhibitors of *Mtb*MetRS; however, additional *in vitro* and *in vivo* investigations are necessary to confirm these findings.

## Graphical abstract

This paper provides a comprehensive approach using the ChEMBL database to construct a machine learning model that screened 10 million compounds from the eMolecules database. Two promising inhibitors targeting *Mycobacterium tuberculosis* methionyl‐tRNA synthetase were identified. The methodology integrates machine learning, virtual screening, molecular docking, MD simulations, MM/PBSA, and DFT analysis.

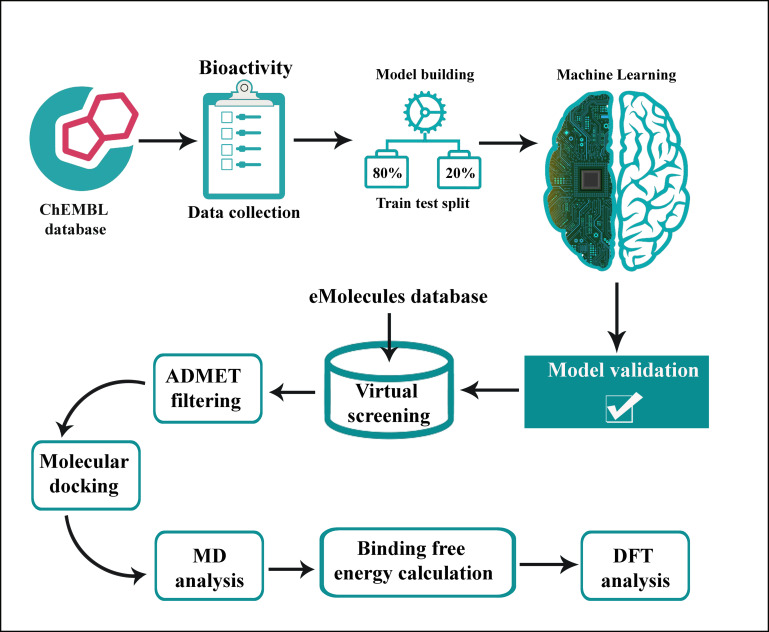

## Introduction

Tuberculosis (TB), one of the oldest infectious disease triggered by *Mycobacterium tuberculosis (Mtb)*, is ranked within the top ten deadliest illnesses globally.^[1]^ TB became the second leading cause of death following the COVID‐19 pandemic,^[2]^ with 1.5 million fatalities in 2020. This data reached to 1.6 million in 2021 and to 1.8 million in 2022. It stays as one of the formidable infectious diseases globally, reflecting the ongoing difficulty in controlling the epidemic.^[3]^

Drug‐susceptible TB can be addressed with first‐line drugs; however, the lengthy treatment time and side effects often lead patients to stop their therapy early. This non compliance increases the risk of developing and spreading of strain of *Mtb* that are resistant to multiple drugs (MDR) and extensively resistant to treatment (XDR).^[4]^ Management of MDR‐TB, alongside XDR‐TB, is complex because it requires long‐term use of several drugs and is difficult for patients to tolerate. This often leads to incomplete treatments that can create resistance to TB drugs. Although newer drugs like bedaquiline, delamanid, and linezolid promise to improve treatment success, drug‐resistant cases have been reported.^[5]^ This highlights the critical need of ongoing research to discover TB drugs.

Experimental high‐throughput screening is expensive and time‐consuming. Artificial intelligence and machine learning (ML) are revolutionizing pharmaceutical discovery and development. These technologies enable efficient screening of large compound libraries, predict drug efficacy and safety, and reduce the cost and time.[^6^, ^7^] To discover novel bioactive molecules targeting *Mtb*, *in silico* methods are often used for screening chemical databases.^[8]^ ML has expanded the scope of this approach to tackle various scientific challenges.^[9]^ Kouchaki et al.^[10]^ utilized a dataset of 13,402 isolates to train their models for stability prediction on both familiar and new samples. They employed three fundamental ML classifiers in accordance with the reduce feature space, along with three ensemble learning techniques. Shahab et al.^[11]^ used different ML models with 10‐fold cross‐validation to identify the top hits compounds from zinc database for thymidylate kinase medication target. ML classification models are based on the principle that the selected biomolecules exhibit common structural characteristics reflecting their biological activities.^[12]^ The ML algorithms can predict molecular properties and interactions with high accuracy, accelerating the initial stages of drug development. Methionyl‐tRNA synthetase (MetRS) is a crucial enzyme in the protein synthesis pathway of all organisms. It facilitates the attachment of methionine to the corresponding tRNA, a fundamental step in initiating protein translation. MetRS is crucial for maintaining bacterial growth and survival, offering a valuable target for drug development. Inhibiting its function could impair protein synthesis and effectively limit bacterial growth, making it a potential strategy for combating TB.^[13]^ The catalytic domain of *Mycobacterium tuberculosis* methionyl‐tRNA synthetase (*Mtb*MetRS) is primarily composed of an α /β structure, extending from the protein's N‐terminus and reaching residue His290. This domain is interrupted by a connective peptide (CP) spanning residues Ile116 to Tyr226.^[13]^ The CP has two regions: CP1, consisting of antiparallel β ‐strands β 4 and β 8, and CP2, consisting of helices α 5 and α 6, as depicted in Figure S1.

Molecular docking is a computationally useful technique that is extensively applied in drug discovery by predicting protein‐ligand interactions and binding affinites.[^14^, ^15^] It utilizes a scoring function to assess the strength of the binding affinity across two compounds based on their preferential orientation.^[16]^ AutoDock 4.2.6 employs Lamarckian genetic algorithm for studying the protein‐ligand conformations on the basis of the most favorable binding energies.^[17]^ The integration of molecular dynamics (MD) simulations with molecular docking enhances the reliability of predicted protein‐ligand complex.^[18]^ These are valuable tools for drug discovery, offering conformational stability, structural motion, and protein‐ligand interactions.[^19^, ^20^]

Density functional theory (DFT) is a computationally efficient method used to predict the structural and electronic properties of drug candidates.^[21]^ The energy difference in the frontier molecular orbitals elucidates the reactivity of the compound.^[22]^ Electrons in the HOMO orbital are loosely bound to the molecule, that makes it easier to displace electron and create a positively charged molecule, whereas LUMO orbitals readily accept electrons.^[23]^ Despite advancements in computational drug discovery, there remains a need for comprehensive studies that integrate multiple computational techniques to validate and optimize potential drug candidates.

This research aims to identify the potential inhibitors of the *Mtb* target by screening the compounds from a large database using ML algorithms, molecular docking, and MD simulations. ADMET and DFT analyses are also performed to ensure pharmacokinetic properties, toxicity profiling, and charge transfer phenomena in bio‐activity. Moreover, the post‐MD free energy of the protein‐ligand system is calculated using the molecular mechanics Poisson‐Boltzmann surface area method (MM/PBSA).

## Methods

### Data Sets Collection and Curation

In this study, 2,993 compounds related to the *Mtb* targets were obtained in SMILES format from the ChEMBL database.^[24]^ The compounds with duplicate SMILES and SMILES lacking IC50 were removed, resulting in 2154 compounds. The IC50 values of compounds were converted into pIC50 values. The dataset was divided into 1070 active and 1084 inactive compounds based on a pIC50 cut‐off value of 6, resulting in a nearly balanced distribution of labels. The close ratio of active to inactive compounds minimizes the risk of data imbalance, which could otherwise negatively affect the reliability and robustness of the models. Here, we selected Lipinski and PaDEL descriptors due to their established relevance in drug discovery, specifically their ability to capture essential physicochemical and topological features critical for understanding small molecule activity. Lipinski descriptors indicate the physical and chemical attributes of the molecules, and they are used to predict the oral activity of drugs. The Lipinski rule of five (LRo5) includes the maximum values of molecular mass (500 Daltons), hydrogen bond donors (5), hydrogen bond acceptors (10), and octanol‐water partition coefficient (5).^[25]^ PaDEL descriptors calculate 797 molecular descriptors, including 663 one‐dimensional, 114 two‐dimensional, and 20 three dimensional descriptors, and fingerprints.^[26]^ Additionally, an external library of more than 10 million compounds was retrieved from the eMolecules database for virtual screening.^[27]^

### Classification and Predictive Model

Curated and preprocessed datasets were utilized to develop quantitative structure‐activity relationship (QSAR) models. These models demonstrate the connection between molecular structure and biological properties and were employed to predict the bioactivity of compounds using Lipinski descriptors and PaDEL descriptors.^[28]^ The dataset was divided in a 4 : 1 ratio for training and testing datasets.^[29]^ Twenty‐seven machine learning classifiers with Scikit‐learn were used to perform QSAR classification.^[28]^ Voting classifier is an ensemble technique that combines the predictions from numerous classifiers to amplify overall performance and robustness. In this study, we used a soft voting approach, where the predicted probabilities from the top three classifiers are averaged, and the class with the highest probability is selected. The hyperparameters of the leading three models were optimized using fivefold cross‐validation approach and combined to create a voting classifier model.^[30]^ Further, to determine the performance of the models produced by each algorithm, metrics like accuracy, specificity, and sensitivity were assessed based on the confusion matrix.[^28^, ^31^] True positive (TP) accurately identifies active inhibitors; true negative (TN) accurately identifies inactive compounds. False positive (FP) mistakenly assign as active and false negative (FN) incorrectly classified as inactive, respectively. A receiver operating characteristic curve (ROC) was generated, and its related area under the curve (AUC) was assessed to evaluate the performance of the classifiers^[32]^ using Eqs. (1)–3.

(1) $$\mathrm{Sensitivity}=\frac{\mathrm{TP}}{\mathrm{TP}+\mathrm{FN}}$$

(2) $$\mathrm{Specificity}=\frac{\mathrm{TN}}{\mathrm{TN}+\mathrm{FP}}$$

(3) $$\mathrm{Accuracy}=\frac{\mathrm{TP}+\mathrm{TN}}{\mathrm{TP}+\mathrm{TN}+\mathrm{FP}+\mathrm{FN}}$$

### ADMET Analysis

Pharmacokinetic properties, like absorption, distribution, metabolism, and excretion of compounds were predicted from pkCSM^[33]^ and SwissADME^[34]^ web servers. Hepatotoxicity and toxicity levels (LD_50_) were determined from ProTox‐II.^[35]^ Additional toxicity parameters (mutagenicity, irritancy, reproductive toxicity, and tumorigenicity) were assessed via Data Warrior software.[^36^, ^37^]

### Protein‐Protein Interactions

The Search Tool for the Retrieval of Interacting Genes/Proteins (STRING) database (version 12.0)^[38]^ was used to performed protein‐protein interaction (PPI) and gene coexpression analysis of *Mtb*MetRS. The protein name “Methionyl‐tRNA synthetase” and the organism *Mycobacterium tuberculosis* H37Rv were entered into the STRING server. A high‐confidence interaction threshold (≥0.9) was applied, with a maximum of 20 proteins selected for interaction analysis. The resulting network was visualized to highlight both direct and indirect interactions of MetRS with other essential proteins. Furthermore, the gene coexpression was predicted in *Mtb* H37Rv and other organisms.

### Molecular Docking

The three‐dimensional structure of *Mycobacterium tuberculosis* methionyl‐tRNA synthetase (*Mtb*MetRS) (PDB ID: 6AX8, resolution: 2.60 Å) was collected from the rcsb protein data bank. The gaps in the residues sequence of the protein structure were built with PyMOL,^[39]^ modelled via modloop,[^40^, ^41^] and prepared after the removal of bound ligand, methionyl‐adenylate (ME8) and other heteroatoms.^[39]^ Grid box was adjusted to 70×70×70 Å with grid center X=271.769, Y=204.067, and Z=1.289 Å, that provides coverage of active amino acid residues Ala9, Ile10, Ala11, Tyr12, His18, Gly20, His21, Tyr23, Glu24, Asp49, Trp228, Ala231, Leu232, Tyr235, Gly261, Asp263, Ile264, His268, Phe292, and Leu293.^[13]^ The bound co‐crystallized ligand was redocked to confirm the docking process. The ME8 from *Mtb*MetRS was extracted and redocked to the catalytic site using AutoDock 4.2.6. The docking procedure is said to be reliable if the root mean square deviation (RMSD) of the original configuration of co‐crystallized ligand and the re‐docked ligand is less than 2 Å.[^42^, ^43^] The semi‐empirical scoring function and the Lamarckian genetic algorithm^[44]^ were used in AutoDock to perform docking on 169 filtered compounds. The search parameter was adopted as the Genetic algorithm, and other parameters were used as default to generate ligand conformational spaces with 100 poses and a population size of 300. The top two candidate compounds were identified based on the docking score, hydrogen bonds and conformations in the cluster. The best binding pose of protein‐ligand complexes was visualized via Protein‐Ligand Interaction Profiler (PLIP)^[45]^ and PyMOL.^[46]^

### Molecular Dynamics Simulations

MD simulations of the best orientation of protein‐ligand complexes were carried out in the GROMACS,^[47]^ version 2022.5. The CHARMM36 force field was employed for protein topology.^[48]^ The online webserver SwissParam was used to generate ligand topology.^[49]^ A periodic box of a dodecahedron was generated outside the protein‐ligand complex, and the simple point charge (SPC) water model was used as the solvent model, with a gap of 10 Å separating solute from the edge of the box.[^50^, ^51^, ^52^] The total charge on the system was balanced by incorporating the appropriate number of Na^+^ and Cl^−^ ions. All the systems were energetically minimized using steepest descent algorithms. The system was equilibrated using NVT/NPT for 100 picosecond (ps), applying the leap‐frog integrator. A modified Berendsen thermostat was utilized to control temperature and isotropic Berendsen pressure coupling was employed to maintain pressure. The linear constraints (LINCS) algorithm was employed to constrain the covalent bonds with hydrogen atoms. The value of fast fourier transform (FFT) grid spacing was set to 0.16 nanometer (nm), and the short range van der Waals cutoff of 1.20 nm was used.^[53]^ Finally, the systems were run for 200 nanosecond (ns) molecular dynamics simulations with a timestep of 2 femtosecond (fs) and a temperature of 300 K. The snapshots of trajectories from 0 to 200 ns were collected at every 100 ps.^[54]^ Employing trajectories acquired from MD simulations, root mean square deviation (RMSD), root mean square fluctuation (RMSF), radius of gyration (R_g_) and solvent accessible surface area (SASA) were studied for conformational changes, compactness, and stability in the apo form and protein‐ligand complexes.

### MM/PBSA Analysis

The binding free energy of both protein‐ligand complexes was calculated using gmx_MMPBSA tool of GROMACS software.^[55]^ The binding free energy of the complex (${\Delta G}_{\mathrm{binding}}$ ) was estimated using Eq. 4.

(4) $${\Delta G}_{\mathrm{binding}}=G_{\mathrm{complex}}-G_{\mathrm{receptor}}-G_{\mathrm{ligand}}$$

where G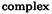 is the binding free energy of receptor‐ligand complex, G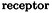 , and G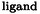 are the unbound free energies of receptor and ligand, respectively. The snapshots were taken from the last 20 ns MD simulations, which were extracted at the intervals of 100 ps with one‐step calculation.

### DFT Calculations

Density functional theory (DFT) is utilized to predict the bioactivity of compounds with the study of their reactivity, molecular structures, and interactions with biological targets. The molecules were optimized employing DFT method with Gaussian 16 W package,^[56]^ using the B3LYP with basis set 6‐311++G(d,p).[^57^, ^58^] The electrical properties, dipole moment, HOMO‐LUMO properties, and molecular reactivity were studied using gas‐phase optimized structures.^[59]^ Moreover, UV‐Vis spectra and density of states were obtained using time‐dependent density functional theory (TD‐DFT) method.^[60]^ The structural visualization, calculation results, and analysis of the molecules were studied with the GaussView 6 software,^[61]^ and GaussSum 3.0.^[62]^ The various parameters of global reactivity descriptors, including electron affinity (A ), ionization potential (I ), chemical softness (β ), chemical hardness (η ), electronic chemical potential (μ ), and global electrophilicity index (ω )[^63^, ^64^, ^65^] of L1 and L2 were evaluated by employing Eqs. (5) and 6.

(5) $$I=E_{\mathrm{HOMO}},\;A=-E_{\mathrm{LUMO}},\;\eta =\frac{1}{2}\left(I-A\right)$$

(6) $$\beta =\frac{1}{\eta },\;\mu =-\frac{1}{2}\left(I+A\right),\;\omega =\frac{\mu^{2}}{2\eta }$$

## Results and Discussion

### Lipinski and PaDEL Descriptors

The performance of machine learning classifiers applied to the collected dataset is presented in Table 1. The accuracy, ROC‐AUC, and F1‐score are calculated for each classifier utilizing both descriptor sets. LabelSpreading is identified as the most precise classifier for Lipinski descriptors, achieving an accuracy of 75 %. However, the Extra Trees Classifier has been found with an accuracy of 89 % when using PaDEL descriptors. PaDEL descriptors are more effective for classification, as indicated by the evaluation metrics. It categorizes the task more effectively than Lipinski by generating a significantly larger number of molecular features.^[66]^

**Table 1:** Performance of machine learning classifiers on test data with default setting.

	Lipinski descriptors			PaDEL descriptors		
Model	Accuracy	ROC‐AUC	F1‐Score	Accuracy	ROC‐AUC	F1‐Score
**LabelSpreading**	0.75	0.75	0.75	0.74	0.68	0.71
**LabelPropagation**	0.74	0.74	0.74	0.74	0.68	0.71
**ExtraTreesClassifier**	0.74	0.74	0.74	0.89	0.89	0.89
**RandomForestClassifier**	0.73	0.72	0.72	0.89	0.88	0.89
**NuSVC**	0.72	0.72	0.72	0.89	0.88	0.88
**KNeighborsClassifier**	0.72	0.71	0.72	0.87	0.87	0.87
**LGBMClassifier**	0.71	0.70	0.70	0.88	0.88	0.88
**XGBClassifier**	0.71	0.70	0.70	0.88	0.88	0.88
**DecisionTreeClassifier**	0.68	0.67	0.67	0.86	0.87	0.86
**ExtraTreeClassifier**	0.67	0.67	0.67	0.88	0.88	0.88
**BaggingClassifier**	0.66	0.66	0.66	0.87	0.87	0.87
**SVC**	0.66	0.65	0.65	0.88	0.87	0.88
**AdaBoostClassifier**	0.62	0.61	0.61	0.81	0.79	0.80
**QuadraticDiscriminantAnalysis**	0.60	0.59	0.59	0.69	0.62	0.65
**GaussianNB**	0.53	0.52	0.53	0.52	0.59	0.46
**LinearDiscriminantAnalysis**	0.53	0.52	0.48	0.86	0.85	0.86
**LogisticRegression**	0.53	0.52	0.48	0.86	0.85	0.86
**RidgeClassifier**	0.53	0.51	0.47	0.86	0.85	0.86
**LinearSVC**	0.53	0.51	0.47	0.85	0.84	0.85
**RidgeClassifierCV**	0.53	0.51	0.47	0.86	0.85	0.86
**CalibratedClassifierCV**	0.53	0.51	0.47	0.86	0.84	0.86
**DummyClassifier**	0.52	0.50	0.36	0.60	0.50	0.45
**BernoulliNB**	0.52	0.50	0.36	0.67	0.68	0.67
**SGDClassifier**	0.48	0.49	0.46	0.84	0.84	0.84
**NearestCentroid**	0.48	0.49	0.47	0.65	0.67	0.66
**PassiveAggressiveClassifier**	0.48	0.47	0.46	0.85	0.85	0.85
**Perceptron**	0.46	0.45	0.43	0.84	0.84	0.84

### Model Building, Performance Evaluation and Cross‐Validation

**Figure 1:**
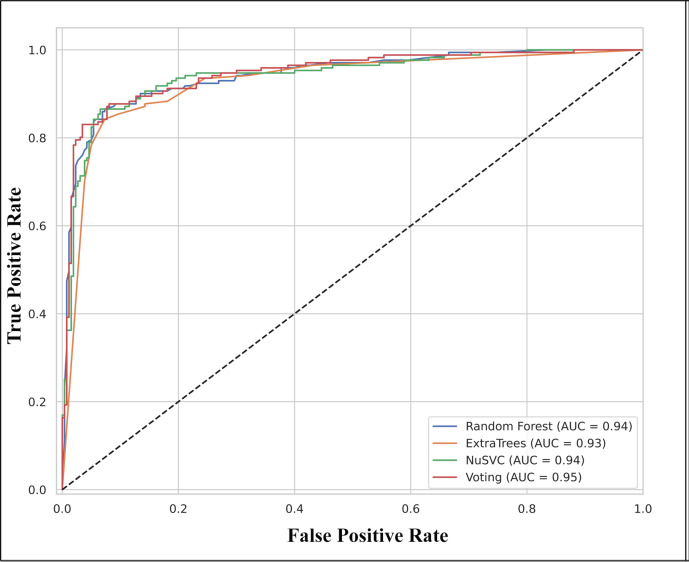
Receiver Operating Characteristic (ROC) curve for each model, with voting classifier exhibiting high accuracy.

**Figure 2:**
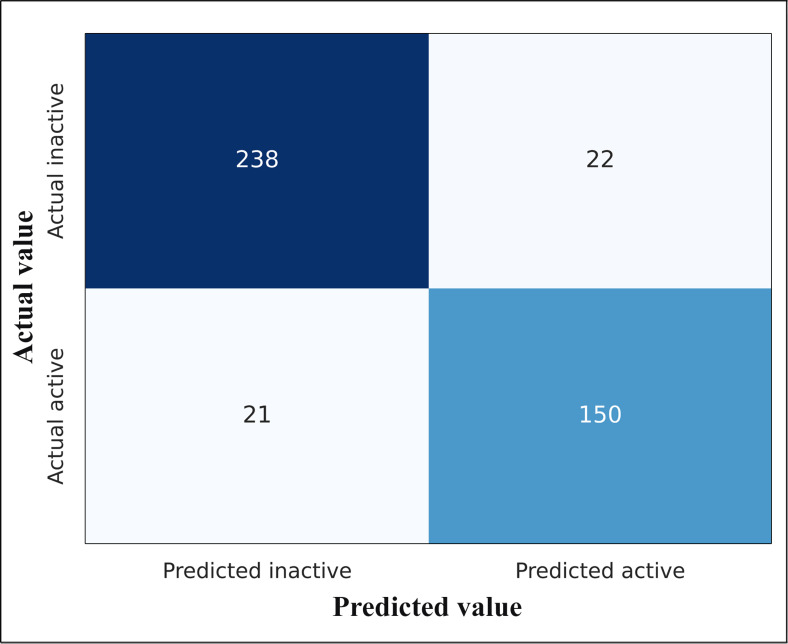
Confusion matrix for voting classifier.

The classifiers Random Forest (RF), Extra Trees Classifier (XT), and Nu‐Support Vector Classification (Nu‐SVC), with the highest scores while employing their default parameters for both descriptors, were used to generate a Voting classifier (VC) model. Hyperparameters were adjusted to identify the most appropriate model parameters for each classification. The criterion was assigned as “gini”, max_features was set to “sqrt”, and n_estimators=100 for RF. For the XT model, n_estimators was configured to 10. For Nu‐SVC, nu was set to 0.2, kernel as “rbf”, and gamma=0.01 and other parameters were set to default. The VC model was generated by combining these top three models with a soft voting method. The effectiveness of the model was examined with the evaluation of accuracy, sensitivity, specificity and the area under the receiver operating characteristic (ROC) curve (AUC) and is depicted in Table 2. The ROC curve provides a graphical representation for evaluating the effectiveness of various models. It is used as a precise metric to evaluate the total performance of different models, indicating better performance for greater AUC values. The three different models (RF, XT, Nu‐SVC), and the voting classifier evaluates the AUC scores of 0.94, 0.93, 0.94, and 0.95 respectively, and are depicted in Figure 1 and Figure S2. The higher value of AUC in VC suggests that this model has good classification performance. The confusion matrix of VC in prediction on the test set for each model is depicted in Figure 2 and Figure S3.

**Table 2:** Evaluation of three individual models and voting classifier on test data with hyperparameters setting.

Model	Accuracy	Sensitivity	Specificity	AUC
RF	0.90	0.86	0.93	0.94
XT	0.89	0.86	0.93	0.93
Nu‐SVC	0.89	0.86	0.91	0.94
VC	0.90	0.88	0.92	0.95

The 10 % discrepancy between training and test accuracy is due to the complexity and real‐world nature of the dataset, which includes important outliers to ensure practical applicability. The high‐dimensional nature of the training features inherently increases the complexity of model. We have prioritized the AUC score to assess the model's performance, attaining a value of 0.95 for VC on test set. This score effectively measures the model's ability to distinguish true positives from false positives, underscoring its predictive power. We have further ensured the robustness and generalizability of the model by employing fivefold cross‐validation. Additionally, testing on an external dataset of reference compounds as shown in Table S3, has demonstrated the model's ability to accurately identify active compounds, with all prediction probabilities exceeding 0.8. These results collectively confirm the model's robustness and its ability to generalize effectively to unseen data.

The fivefold cross‐validation results are presented in Table 3 with the random selection of 20 % of the data. The AUC value of each model suggests that the models are capable of predicting classification. The VC model was selected to filter the eMolecules database. A total of 590 molecules were identified as active with a prediction probability greater than 0.9. Finally, 169 molecules were prepared for molecular docking after screening these 590 active molecules based on mutagenicity, irritancy, reproductive toxicity, and tumorigenicity using DataWarrior Software.

**Table 3:** Five fold cross‐validation of three individual models and voting classifier.

Model	Accuracy	Sensitivity	Specificity	AUC
RF	0.88±0.02	0.82±0.04	0.92±0.02	0.95±0.01
XT	0.87±0.02	0.86±0.01	0.92±0.01	0.93±0.01
Nu‐SVC	0.88±0.02	0.83±0.04	0.91±0.01	0.94±0.01
VC	0.88±0.02	0.82±0.04	0.92±0.02	0.95±0.01

### Protein‐Protein Interactions

**Figure 3:**
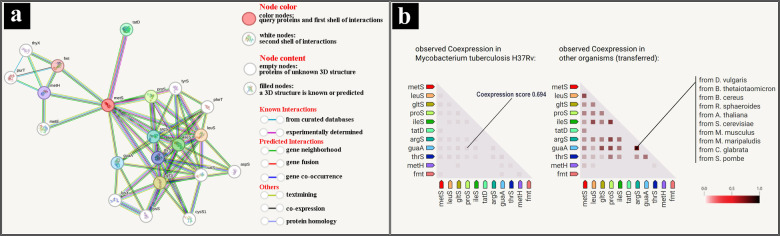
(a) Protein interaction network generated from STRING database, and (b) heat map of gene coexpression analysis.

STRING analysis has identified *Mtb*MetRS as a central component of a PPI network crucial for bacterial survival. Several aminoacyl‐tRNA synthetases, such as leucyl‐tRNA synthetase (LeuS), prolyl‐tRNA synthetase (ProS), isoleucyl‐tRNA synthetase (IleS), glycyl‐tRNA synthetase (GltS), and threonyl‐tRNA synthetase (ThrS) are involved in the aminoacylation process to ensure the translational fidelity during protein synthesis, which is depicted in Figure 3(a). Additional interactions are observed with methionine synthase (MetH) and formyltransferase (Fmt), emphasizing the involvement of MetRS in methionine biosynthesis and translational initiation. The disruption of MetRS effects on protein synthesis and metabolic pathways, thereby posing a significant threat to mycobacterial survival. These findings highlight the therapeutic potential of targeting MetRS, as its inhibition could critically impair bacterial growth and survival pathways. Furthermore, the coexpression of genes was analyzed as a heat map shown in Figure 3(b) where the light color square boxes in left side indicate the gene coexpression levels in *Mtb* H37Rv. The coexpression scores between MetS (or MetRS) and LeuS, GltS, ProS, IleS, TatD, ArgS, GauS, ThrS, MetH, and Fmt are 0.667, 0.354, 0.424, 0.281, 0.364, 0.340, 0.421, 0.239, 0.146, and 0.042, respectively. Additionally, the observed coexpressions in other organisms are shown on the right side of the Figure 3(b).

### Conservation Analysis

**Figure 4:**
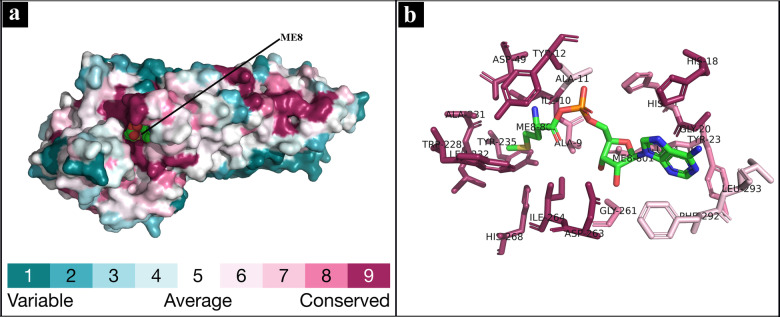
Conservation of amino acid residues in the active site of *Mtb*MetRS: (a) surface representation together with cofactor methionyl‐adenylate (ME8), colored by atom type with sphere structure, and (b) amino acid residues colored by conservation grade and shown in sticks with ME8.

The conservation analysis of the target protein (*Mtb*MetRS) structure was performed using ConSurf tool,^[67]^ which retrieves homologous sequences and calculates conservation scores based on phylogenetic relationships. This automated process enable efficient identification of functionally relevant residues. As shown in Figure 4, the amino acid residues have been categorized based on their conservation levels using the ConSurf color scale: highly conserved (dark purple), moderately conserved (light purple), variable (white), and highly variable (turquoise).^[68]^ The residues Ala11, Phe292, and Leu293 are classified as moderately conserved (color scale 7), suggesting their potential role in maintaining structural integrity through hydrophobic interactions. In contrast, residues Ala9, His21, Tyr23, Glu24, and Gly261, with higher conservation (color scale 8), play critical functional or structural roles. Highly conserved residues, including Ile10, Tyr12, His18, Gly20, Asp49, Trp228, Ala231, Leu232, Tyr235, Asp263, Ile264, and His268 (color scale 9), are crucial for maintaining protein stability and facilitating the ligand binding interactions.

### Molecular Docking

**Figure 5:**
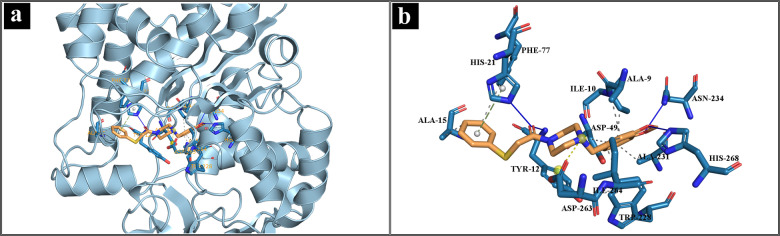
(a) *Mtb*MetRS‐L1 complex with ligand in cartoon view, and (b) interaction of active amino acid residues with L1.

The molecular docking method employed in this study was set up by re‐docking the co‐crystallized ligand. The ME8 inhibitor from *Mtb*MetRS was extracted and docked again into the binding site using AutoDock 4.2.6. It was evaluated based on RMSD value. The RMSD of co‐crystallized ligand and re‐docked ME8 was found to be 1.644 Å with docking score of −5.96 kcal/mol, as depicted in Figure S5. The active amino acid residues Ala9, Ile10, Tyr12, His18, Gly20, His21, Glu24, Asp49, Trp228, Ala231, Tyr235, Gly261, Asp263, Ile264, His268, Phe292, and Leu293 are responsible for the functioning of enzymes and form the binding pocket.^[13]^ The reference drug linezolid (Lin) (CID 441401) has the binding score of −8.07 kcal/mol. Tyr12, His21, Lys54, Asp263, and His268 are involved in the formation of hydrogen bonds. Furthermore, Ala9, Tyr12, Ile264 are involved in hydrophobic interactions, as depicted in Figure S6. The ligand L1 (CID 4807041) has the highest binding score of −12.74 kcal/mol. It forms hydrogen bonds with Tyr12, His21, Asn234, and His268 in the active site. Additionally, Ala9, Ile10, Ala15, Asp49, Phe77, Trp228, Ala231, and Ile264 are involved in hydrophobic interactions, which is depicted in Figure 5. Furthermore, His21, and Asp263 are associated with π ‐stacking and salt bridges, respectively.

It is observed that *Mtb*MetRS and ligand L2 (CID 3751463) form three hydrogen bonds with Tyr12, Leu293, and Lys299 with docking score of −11.83 kcal/mol. Amino acid residues Tyr12, Trp228, and Phe292 are involved in the hydrophobic interactions, as depicted in Figure S7. Additionally, His18, and His294 are associated with π ‐stacking and salt‐bridges respectively. Therefore, L1, and L2 indicated potent interactions with the residues introduced at the ME8 binding site.

To analyze the structural relationships between the reference drug Lin and the top five compounds (L1 to L5), Morgan2 fingerprint similarity has been utilized as shown in Figure S8, where K_i_ represents the inhibition constant obtained by molecular docking targeting *Mtb*MetRS. Tanimoto and Dice similarity coefficients are evaluated based on Morgan and MACCS fingerprints, depicted in Table S4. The results of molecular docking and dynamics have also been compared for Lin, L1 and L2 in complex with *Mtb*MetRS.

### Molecular Dynamics Simulations

Molecular docking captures only single snapshots of receptor‐ligand interaction. MD simulations represents molecular interactions in all simulated frames, as in biological systems.^[69]^ They were carried out on *Mtb*MetRS‐Apo (Apo), *Mtb*MetRS‐L1 (P‐L1), *Mtb*MetRS‐L2 (P‐L2) and *Mtb*MetRS‐Lin (P‐Lin) complexes to understand their structural changes at atomic‐level, stability, and flexibility.

#### Root Mean Square Deviation (RMSD)

**Figure 6:**
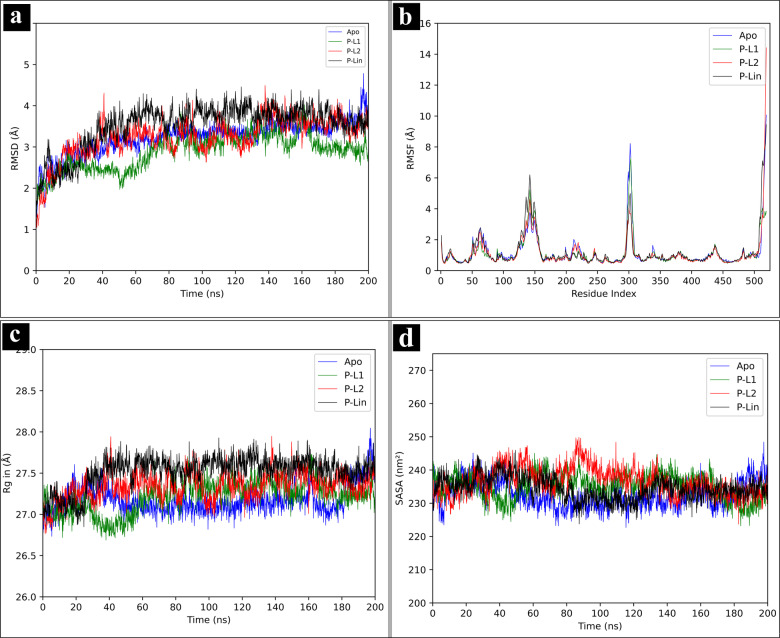
Structural dynamics of *Mtb*MetRS (a) RMSD of the backbone atoms, (b) RMSF, (c) R_g_, and (d) SASA for Apo (blue), P‐L1 (green), P‐L2 (red), and P‐Lin (black) throughout 200 ns.

RMSD measures the modification of protein's structure over time with respect to the initial structure.^[70]^ The average RMSD values of Apo, P‐L1, P‐L2 and P‐Lin are measured with 3.22±0.40 Å, 2.88±0.42 Å, 3.24±0.48 Å, and 3.51±0.53 Å respectively, and are depicted in Figure 6(a). Significantly, P‐L1 has shown the lowest RMSD value, which confirmed its stability compared to Apo, P‐L2 and P‐Lin. Both P‐L1 and P‐L2 attained the equilibration state after 180 ns of MD simulations. These results demonstrate the stability of P‐L1 and P‐L2 complexes, and their binding characteristics.

#### Root Mean Square Fluctuation (RMSF)

RMSF provides information about the flexible regions of the protein, showing the displacement of amino acid residues compared to the reference structure.^[71]^ The coils, loop and turns show higher RMS fluctuation with compared to sheet and helical structures. The overall average RMSF values of Apo, P‐L1, P‐L2, and P‐Lin are 1.16, 1.11, 1.11, and 1.21 Å, respectively. The RMSF of selected two complexes has been compared with the apo form and P‐Lin protein structure. The higher peaks in the Figure 6(b) indicate the fluctuation of residues in loosely arranged loops. Structures like sheets and helices showed lower RMSF values. The results of RMSF in the complexes showed less RMSF values compared to Apo, suggesting that the residues do not fluctuate much after binding. The higher fluctuation regions of protein structure of each complexes are depicted in Figure S9 with cartoon view in respective mesh structures. The RMSF values of active amino acid residues, depicted in Table S5, suggest that fluctuation of active amino acid residues decreased due to ligand binding in active site.

#### Radius of Gyration (R_g_)

R_g_ is used to identify the compactness of receptor‐ligand complex during molecular dynamics simulations.^[72]^ The average R_g_ values obtained for Apo, P‐L1, P‐L2, and P‐Lin complexes are 27.17±0.16 Å, 27.21±0.19 Å, 27.34±0.16 Å, and 27.51±0.19 Å, respectively. All the complexes exhibit nearly constant R_g_ values (~27 Å) over 200 ns of MD simulations as depicted in Figure 6(c). Among all, P‐L1 displayed the lowest R_g_ value, indicating a more compact and stable structure compared to other complexes.

#### Solvent Accessible Surface area (SASA)

SASA measures the surface area of the receptor that is accessible to solvent.^[73]^ The SASA values (average±standard deviation) for Apo, P‐L1, P‐L2, and P‐Lin are determined to be 232.42±3.85 nm^2^, 235.13±3.80 nm^2^, 236.69±4.21 nm^2^, and 234.44±3.41 nm^2^, respectively. Among these, P‐L2 exihibits the highest SASA value, signifying greater exposure of surface area. The lowest SASA value for P‐L1 ranges from 223.20 nm^2^ to 245.81 nm^2^, suggesting a compact structure and stable interaction with the active amino acid residues of *Mtb*MetRS as in Figure 6(d).

#### Hydrogen bonds

Hydrogen bond analysis measures specificity, directionality, and molecular recognition.^[74]^ The number of hydrogen bonds at the active site of *Mtb*MetRS are calculated throughout the 200 ns of MD simulations. These numbers were determined by considering the distance between the donor and acceptor atoms to be less than 3.5 Å. The hydrogen bonds formed in each complex within the total trajectories of 200 ns simulations are depicted in Figure S10. The hydrogen bonds formed in P‐L1 shows greater stability than in P‐L2.

### MM/PBSA Free Energy

**Figure 7:**
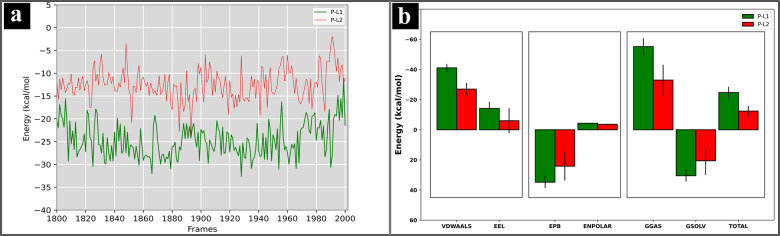
Binding energy profile of last 20 ns for (a) P‐L1 and (b) P‐L2 complex.

The MM/PBSA was performed on both P‐L1 and P‐L2 complexes, extracted from last 20 ns using 200 frames. MM/ PBSA provides the precise prediction of binding energies of complexes and the binding affinities of ligands.^[75]^ The MM/PBSA scores of complexes are provided by van der Waals (VDWAALS), electrostatic (EEL), polar solvation (EPB), and non‐polar solvation energy (ENPOLAR), as depicted in Figure 7(b). The polar solvation energy (P‐L1: 34.82±3.92 kcal/mol and P‐L2: 24.15±9.6 kcal/mol), the electrostatic energy (P‐L1: −14.13±4.46 kcal/mol and P‐L2: −5.99±8.37 kcal/mol) and van der Waals energy (P‐L1: −41.08±2.52 kcal/mol and P‐L2: −26.98±4.1 kcal/mol) contributed significantly to negative binding energies, favoring strong interactions. Consequently, the total binding energies of P‐L1 and P‐L2 complexes are −24.70±3.80 kcal/mol and −12.40±3.41 kcal/mol, respectively. This results indicate that P‐L1 has a stronger binding affinity compared to P‐L2. The energetics of last 20 ns of equilibrated simulations are plotted in Figure 7(a), supporting the stability and binding properties of both complexes. The amino acid residues contributing energy for L1 and L2 to interact with receptor are depicted in Figure S11 and Figure S12. Multiple amino acid residues: Ala9, Ala11, Tyr12, Tyr235 and Ile264 contributed significantly to negative binding energy in P‐L1, suggesting that P‐L1 forms stronger and stable interactions with *Mtb*MetRS target. In P‐L2 complex, Phe292 emerged as the primary contributor to negative binding energy, indicating fewer key interactions with *Mtb*MetRS target, making it potentially less stable compared to P‐L1.

### Density Functional Theory Calculations and Analysis

#### Structural Analysis

**Figure 8:**
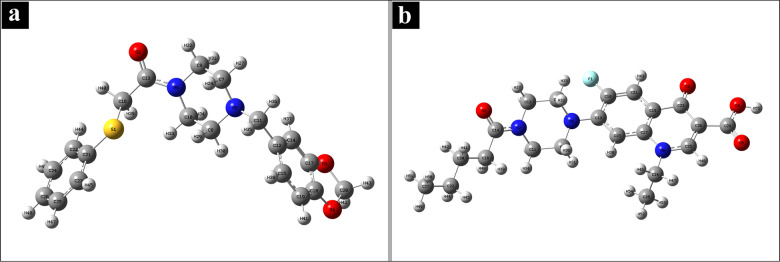
Optimized structures employing DFT at B3LYP/6‐311++G(d,p) level of calculation: (a) L1 and (b) L2.

The optimized structures of L1 and L2 are depicted in Figure 8. The corresponding lowest total energies and dipole moments for both compounds are presented in the Table 4. Notably, the lowest total energy for L1 is −1509.04 Hartrees, which is lower than the corresponding energy of L2, at −1381.31 Hartrees, by 127.73 Hartrees, indicating L1 is more stable. The dipole moment depicts the charge distribution and the localization within the molecule.^[76]^ The higher dipole moments of L2 (8.28 Debye) than L1 (3.93 Debye) suggests that L2 exhibits a greater ability to participate in polar reactions.^[77]^

**Table 4:** Energy and dipole moment for compounds L1 and L2.

Compounds	Total Energy (Hartrees)	Dipole Moment (Debye)
L1	−1509.04	3.93
L2	−1381.31	8.28

#### Frontier Molecular Orbitals

**Figure 9:**
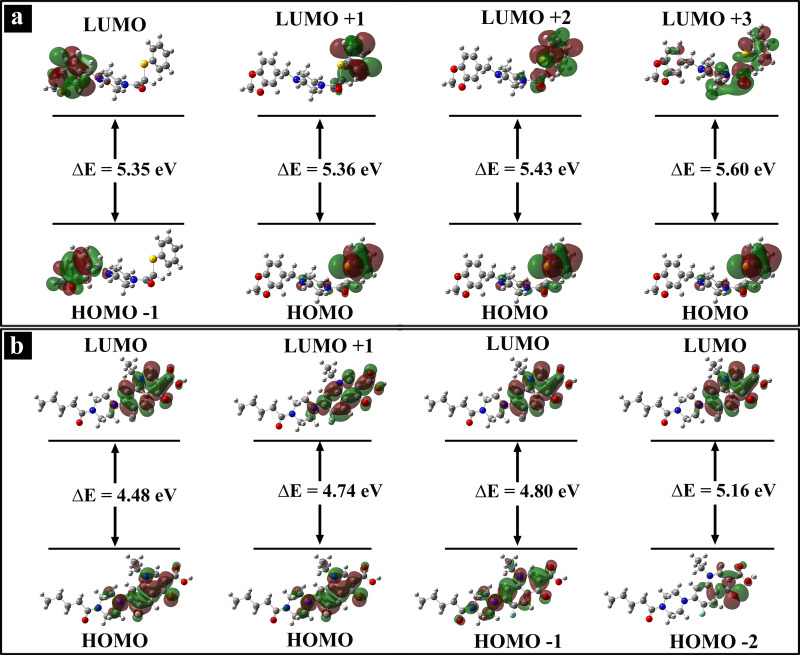
Energy gap of frontier molecular orbitals (a) L1 and (b) L2.

**Figure 10:**
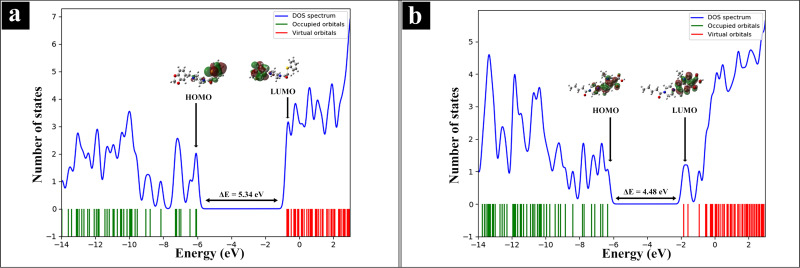
Density of states in occupied and virtual orbitals (a) L1 and (b) L2.

The energies of frontier molecular orbitals are determined using TD‐DFT at the B3LYP/6‐311++G(d,p) level of theory and displayed in Figure 9. The red section around the atoms manifests the positive phases, whereas green section represents the negative phases. The energy gaps between HOMO and LUMO for L1 and L2 are 5.34 eV and 4.48 eV, respectively, and the computed values are in favor with the DOS spectra as in Figure 10. These results show L1 and L2 can take part in charge transfer mechanism and posses potential for bio‐activities.[^78^, ^79^]

**Figure 11:**
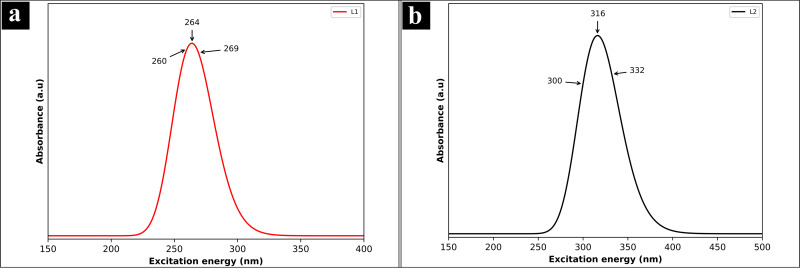
Ultraviolet‐visible spectra, (a) L1 and (b) L2.

The ultraviolet‐visible spectra of L1 and L2 are obtained to elucidate their absorption properties, charge transfer characteristics, and excitation energies. The maximum wavelength of visible absorption, oscillator strength, and major contributions of orbitals are depicted in Table 5. The wavelength 269 nm for L1, and 332 nm for L2 are major contributing for the formation of absorption band, as shown in Figure 11. The absorption maxima at these wavelengths indicate notable electronic transitions within the molecules. Specifically, the lower absorption wavelength of L1 suggests that it may undergo transitions involving less energy compared to L2.^[80]^

**Table 5:** Electronic properties of L1 and L2 with major contribution of orbitals.

Molecule	Calculated (nm)	Oscillatory strength (f )	Orbital description for major contributions
L1	269	0.0121	H→L+1 (84 %)
	264	0.0917	H−1→L (83 %)
	260	0.0414	H→L+2 (35 %)
L2	332	0.0010	H−2→L (81 %)
	316	0.1368	H→L (88 %)
	300	0.0008	H−1→L (42 %)

#### Global Reactivity Descriptors

The corresponding values of different parameters of global reactivity descriptors are listed in Table 6. The compounds are categorized as strong, moderate, and marginal electrophiles for corresponding values of ω greater than 1.5 eV, between 0.8 to 1.5 eV, and less than 0.8 eV, respectively.^[81]^ The evaluated values of electrophilicity for L1 (2.15 eV) and L2 (3.72 eV) indicate strong electrophiles, and the small values of electronic chemical potential suggest that these compounds are able to donate electrons. Furthermore, the chemical softness of L1 and L2 is observed to be 0.37 per eV and 0.44 per eV, respectively. The small value in μ suggests that both L1 and L2 are reactive in nature.^[82]^

**Table 6:** Global reactivity descriptors of L1 and L2 compounds.

Compounds	$E_{\mathrm{HOMO}}(\mathrm{eV})$	$E_{\mathrm{LUMO}}(\mathrm{eV})$	$E_{\mathrm{LUMO}}-$ $E_{\mathrm{HOMO}}(\mathrm{eV})$	A (eV)	I (eV)	η (eV)	β (eV)^−1^	μ (eV)	ω (eV)
L1	−6.06	−0.72	5.34	0.72	6.06	2.67	0.37	−3.39	2.15
L2	−6.32	−1.84	4.48	1.84	6.32	2.24	0.44	−4.08	3.72

#### Molecular Electrostatic Potential

**Figure 12:**
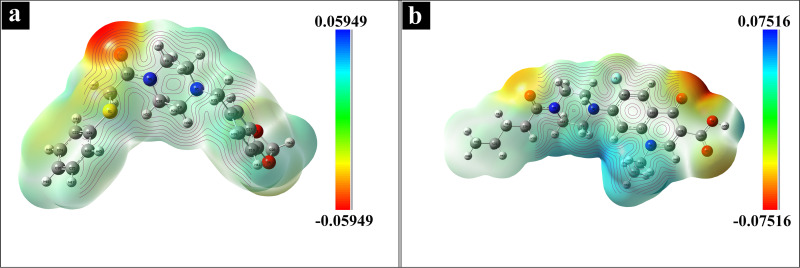
Molecular electrostatic potential (MEP) map of (a) L1 and (b) L2.

Molecular electrostatic potential (MEP) is utilized to map electron distribution within molecules and for identifying potential electrophilic and nucleophilic sites.[^83^, ^84^] The positive potential regions, represented in red, are classified as electrophilic, while the regions of negative potential, shown in blue, are nucleophilic; the areas with zero potential are represented in green. The MEP maps of L1 and L2 are depicted in Figure 12. The comparative positive regions are obtained on hydrogen atoms pointing electrophilic sites, and the maximum negative regions are spotted around oxygen atoms O2 in L1 and O3 in L2, indicating nucleophilic sites. This finding suggests that the hydrogen atoms are likely to participate in electrophilic interactions, while the oxygen atoms act as nucleophiles, facilitating nucleophilic attacks on electrophilic centers within chemical reactions.^[85]^

### ADMET Analysis

The beneficial pre‐clinical phase, in agreement with Lipinski's rule of 5 (LRo5), has mentioned that mouth‐administered drugs should meet at least three criteria. Furthermore, water solubility should be greater than −5, and topological polar surface area (TPSA) should remain under 140 Å^2^.^[86]^ The estimated water solubility values for L1 and L2 are −3.792 and −3.245, respectively. Furthermore, the TPSA values for L1 and L2 are 67.31, and 82.85 Å^2^, respectively. The six parameters – lipophilicity, flexibility, insaturation, insolubility, polarity, and size of L1 and L2 – represented by pink area are in favour of radar plot range, as shown in Figure S13. The bioavailability scores of L1 (0.55) and L2 (0.56) indicate favorable pharmacokinetic properties. The Brain Or IntestinaL EstimateD permeation method (BOILED‐Egg) was employed to estimate the permeability of selected molecules based on their potential for blood‐brain barrier (BBB) and passive human gastrointestinal absorption (HIA). The BOILED‐Egg graph of L1 and L2, along with FDA‐approved drugs like first‐line drug (isoniazid), second‐line drug (ethionamide), and MDR‐TB drug (linezolid), is presented in Figure S14. It indicates that isoniazid, ethionamide, L1, and L2 are not P‐glycoprotein (P‐gp) substrates, whereas linezolid is a P‐gp substrate. All the compounds are predicted to have favourable absorption characteristics, and only L1 is expected to penetrate the blood‐brain barrier. The LD_50_ values of both L1 (750 mg/kg; toxicity class IV) and L2 (4000 mg/kg; toxicity class V) suggest that they exhibit relatively low toxicity.

## Conclusions

In the real‐world applications, machine learning (ML) models are invaluable for the screening of large chemical dataset, significantly reduce the time consumption and necessity of experimental screening. These models also accelerate the discovery of promising drug candidates. The strong correlation between the ML model predictions and the simulation outcomes demonstrates the efficiency of the model in identifying biologically relevant compounds. Incorporating molecular docking and molecular dynamics (MD) simulations as validation steps ensures the practical applicability of the models and reduces the risk of false positives. In our study, the application domain of ML models included the chemical space with molecules following Lipinski's rule of five. The accuracy and reliability of the model's predictions were verified by the simulation outcomes. To identify potential inhibitors against *Mycobacterium tuberculosis* methionyl‐tRNA synthetase (*Mtb*MetRS), we employed the integrated computational techniques such as ML models, molecular docking, MD simulations, ADMET analysis and density functional theory (DFT). A total of 590 molecules were primarily selected from approximately 10 million molecules and filtered down to 169 based on mutagenicity, irritancy, reproductive toxicity, and tumorigenicity. Among these molecules, 1‐[4‐(1,3‐benzodioxol‐5‐ylmethyl) piperazin‐1‐yl]‐2‐phenylsulfanylethanone (L1) and 1‐ethyl‐6‐fluoro‐4‐oxo‐7‐(4‐pentanoylpiperazin‐1‐yl)quinoline‐3‐carboxylic acid (L2) emerged as top candidates with strong docking scores and stable interactions with *Mtb*MetRS, as demonstrated by MD simulations. Moreover, MM/PBSA calculations confirmed the stability of these interactions, with L1 showing a strong binding affinity than L2. DFT calculations further supported the bio‐active nature of these compounds in charge transfer process. Both L1 and L2 demonstrated favorable pharmacokinetic and physicochemical properties. The predicted LD_50_ values of L1 (750 mg/kg) and L2 (4000 mg/kg) indicated that both L1 and L2 have a safer toxicity profile. Therefore, L1 and L2 would be the potential inhibitors against *Mtb*MetRS. Further *in vitro* and *in vivo* studies are recommended for the validation of these findings.

## Author Contributions

Rajesh Maharjan: Conceived and designed the study, performed data analysis, prepared the figures, and wrote the manuscript; Kalpana Gyawali: Critical feed back and revised the manuscript; Arjun Acharya: Technical support, data analysis, critical feed back and revised the manuscript; Madan Khanal: Critical feed back, data analysis and revised the manuscript; Kamal Khanal: Critical feed back, data analysis and revised the manuscript; Mohan Bahadur Kshetri: Critical feed back and revised the manuscript; Madhav Prasad Ghimire: Technical support, critical feedback, revised the manuscript and supervised the research work; Tika Ram Lamichhane: Technical support, critical feedback, data analysis, revised the manuscript and supervised the research work.

## Conflict of Interests

The authors declare that there is no conflict of interest.

## Supporting information

As a service to our authors and readers, this journal provides supporting information supplied by the authors. Such materials are peer reviewed and may be re‐organized for online delivery, but are not copy‐edited or typeset. Technical support issues arising from supporting information (other than missing files) should be addressed to the authors.

Supporting Information

## Data Availability

The data supporting the findings of this study are available within the article and its supplementary materials.
