# Supplementary material for: Machine Learning‐Based High‐Throughput Screening, Molecular Modeling and Quantum Chemical Analysis to Investigate Mycobacterium tuberculosis MetRS Inhibitors
Source: ChemistryOpen. 2025 Feb 25;14(7):e202400460. doi: 10.1002/open.202400460 (PMC12256936; doi:10.1002/open.202400460)
Supplement: Supplementary file 1 — Supporting Information [file OPEN-14-e202400460-s001.pdf]

ChemistryOpen

Supporting Information

Machine Learning-Based High-Throughput Screening, Molecular Modeling and Quantum Chemical Analysis to Investigate *Mycobacterium tuberculosis* MetRS Inhibitors

Rajesh Maharjan, Kalpana Gyawali, Arjun Acharya, Madan Khanal, Kamal Khanal, Mohan Bahadur Kshetri, Madhav Prasad Ghimire, and Tika Ram Lamichhane*

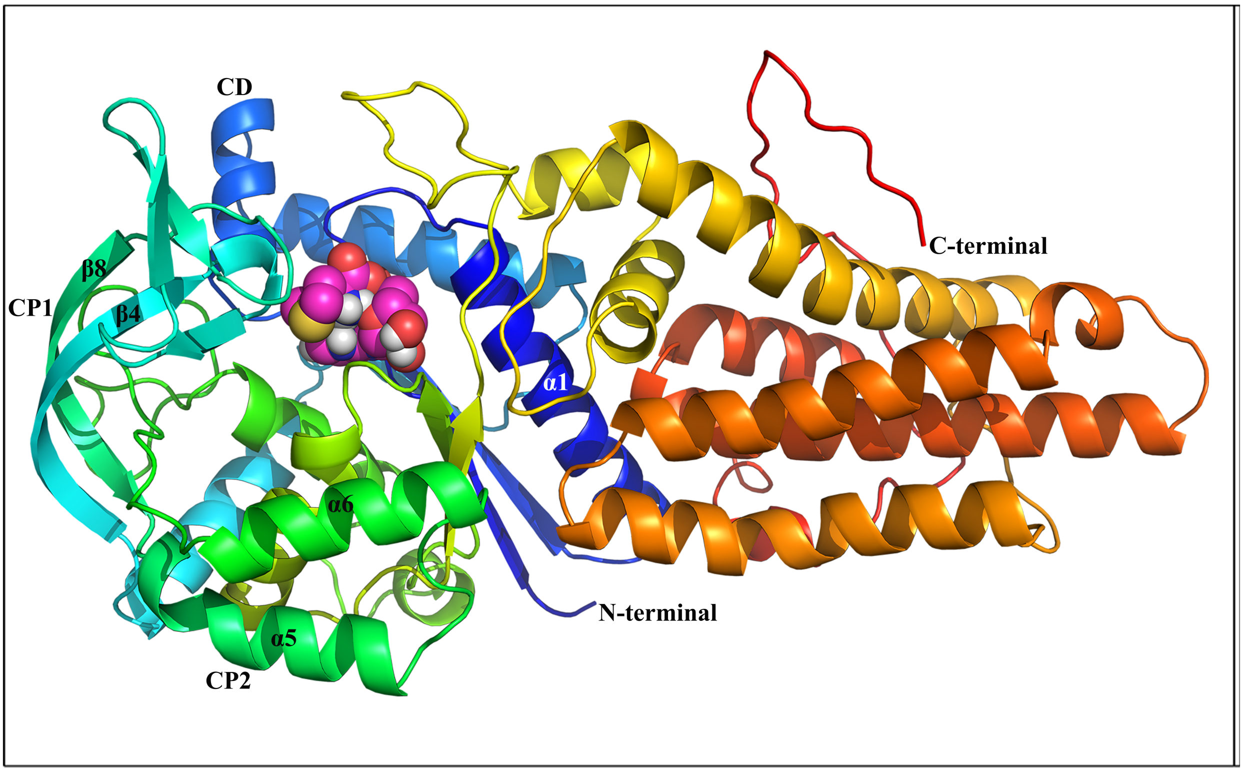

Figure S1: 3D structure of *Mtb*MetRS liganded with ME8. CP1 and CP2 subdomains of connective peptide domain (CP) shown in green. ME8 is shown as spheres with C, H, N, O, S, and P as pink, white, blue, red, yellow, and orange, respectively.

Table S1: **Performance of machine learning classifiers on training data from the ChEMBL dataset**

	**Lipinski descriptors**			**PaDEL descriptors**		
**Model**	**Accuracy**	**ROC-AUC**	**F1-Score**	**Accuracy**	**ROC-AUC**	**F1-Score**
**RandomForestClassifier**	1.00	1.00	1.00	1.00	1.00	1.00
**DecisionTreeClassifier**	1.00	1.00	1.00	1.00	1.00	1.00
**ExtraTreeClassifier**	1.00	1.00	1.00	1.00	1.00	1.00
**ExtraTreesClassifier**	1.00	1.00	1.00	1.00	1.00	1.00
**BaggingClassifier**	0.99	0.98	0.99	0.99	0.99	0.99
**XGBClassifier**	0.97	0.96	0.97	0.99	0.99	0.99
**LGBMClassifier**	0.94	0.93	0.94	0.99	0.99	0.99
**LabelPropagation**	0.92	0.92	0.92	1.00	1.00	1.00
**LabelSpreading**	0.92	0.91	0.92	1.00	1.00	1.00
**KNeighborsClassifier**	0.82	0.82	0.82	0.90	0.90	0.90
**NuSVC**	0.80	0.79	0.80	0.94	0.93	0.94
**AdaBoostClassifier**	0.69	0.67	0.69	0.82	0.80	0.82
**SVC**	0.69	0.65	0.67	0.94	0.93	0.94
**QuadraticDiscriminantAnalysis**	0.61	0.58	0.59	0.70	0.63	0.65
**RidgeClassifierCV**	0.59	0.53	0.54	0.94	0.93	0.94
**LogisticRegression**	0.59	0.53	0.54	0.96	0.96	0.96
**RidgeClassifier**	0.59	0.53	0.54	0.94	0.93	0.94
**GaussianNB**	0.54	0.53	0.54	0.53	0.60	0.48
**LinearSVC**	0.59	0.53	0.54	0.98	0.98	0.98
**LinearDiscriminantAnalysis**	0.59	0.53	0.53	0.94	0.93	0.94
**CalibratedClassifierCV**	0.59	0.53	0.52	0.94	0.93	0.94
**NearestCentroid**	0.51	0.52	0.51	0.66	0.68	0.66
**SGDClassifier**	0.48	0.51	0.47	0.91	0.91	0.91
**DummyClassifier**	0.59	0.50	0.43	0.59	0.50	0.44
**BernoulliNB**	0.59	0.50	0.43	0.67	0.68	0.67
**PassiveAggressiveClassifier**	0.53	0.49	0.51	0.92	0.92	0.92
**Perceptron**	0.52	0.48	0.50	0.89	0.89	0.89

Table S2: **Evaluation of three individual models and voting classifier for training data with hyperparameters setting**

Model	Accuracy	Sensitivity	Specificity	AUC
RF	0.99	0.99	0.99	1.00
XT	0.99	0.99	0.99	1.00
Nu-SVC	0.97	0.95	0.98	1.00
VC	0.99	0.99	1.00	1.00

Table S3: **Bioactive probability of four reference compounds predicted from VC model**

Compounds	Probability
Linezolid	0.85
Bedaquiline	0.86
Pretomanid	0.81
Pyrazinamide	0.81

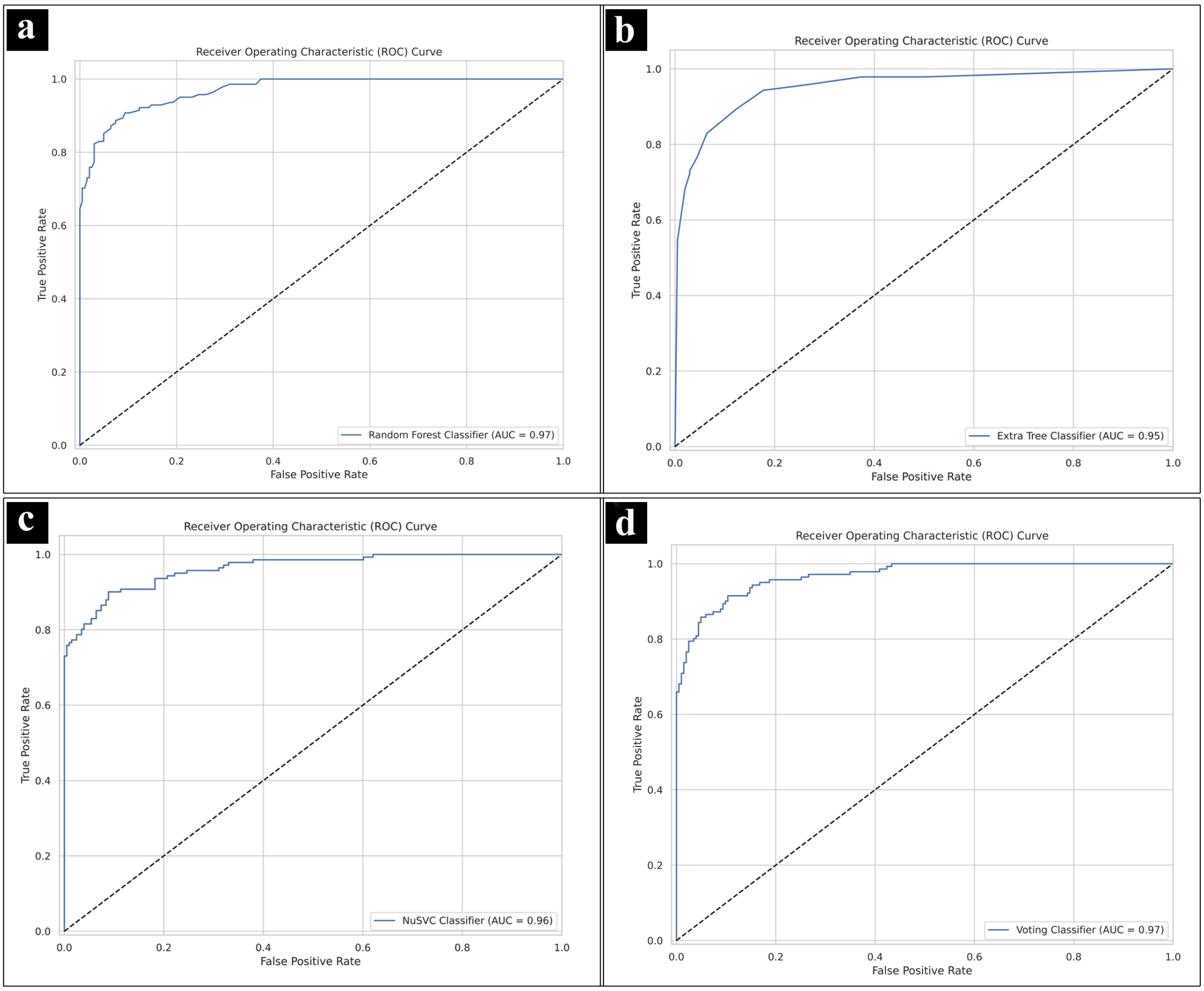

Figure S2: Receiver Operating Characteristic (ROC) curve for (a)Random Forest Classifier, (b) Extra Tree Classifier, (c) Nu-SVC Classifier, and (d) Voting Classifier.

Table S4: Similarity checking between linezolid with the top five compounds using Morgan2 and MACCS fingerprints

reference drug	pubchem_ID	tanimoto_maccs	tanimoto_morgan	dice_maccs	dice_morgan
Linezolid	4807041	0.567568	0.208333	0.724138	0.344828
	3751463	0.706667	0.254237	0.828125	0.405405
	156268	0.693333	0.287037	0.818898	0.446043
	717934	0.538462	0.196581	0.700000	0.328571
	3684314	0.689189	0.290909	0.816000	0.450704

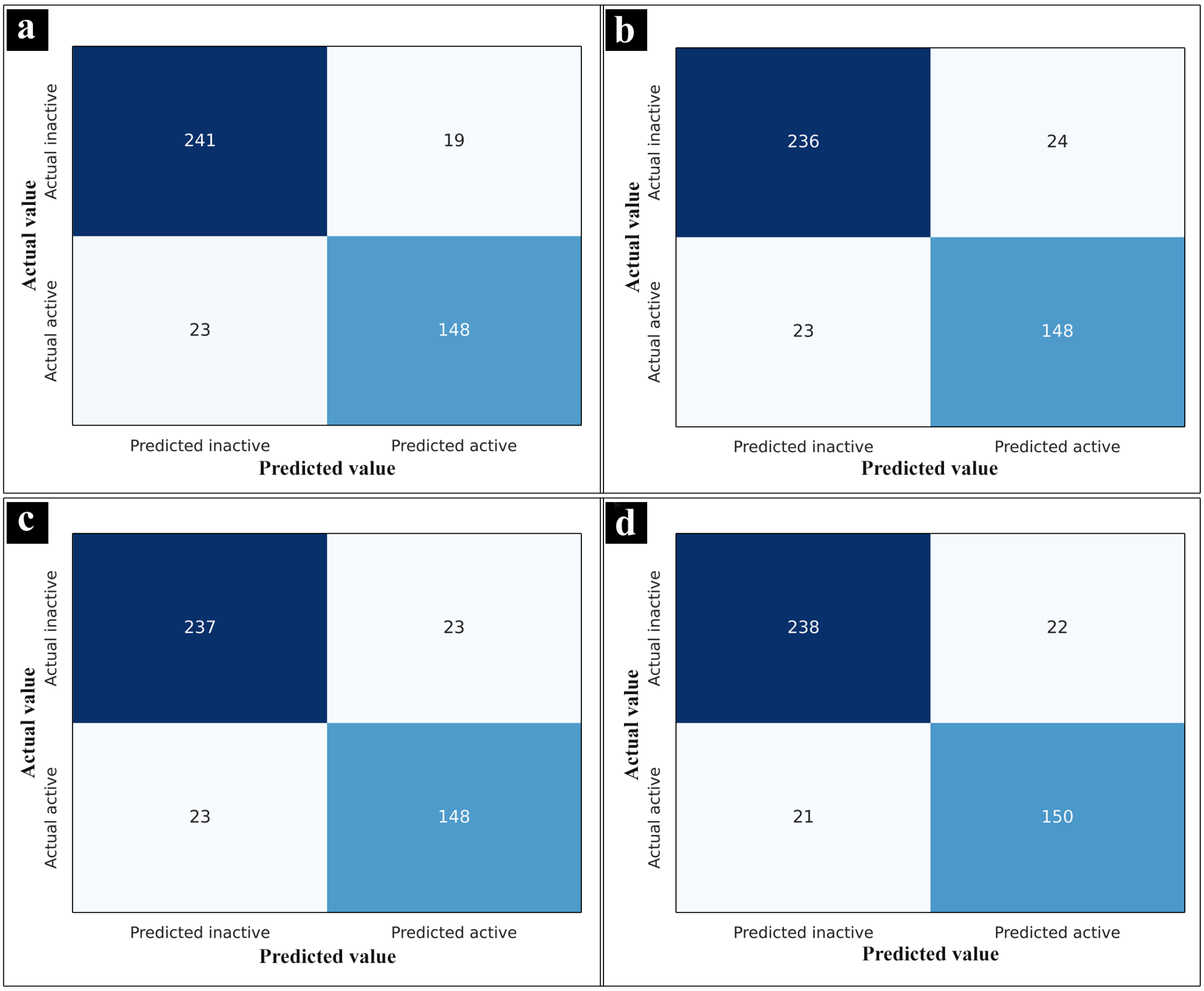

Figure S3: Confusion matrix for (a) Random Forest Classifier, (b) Extra Tree Classifier, (c) Nu-SVC Classifier, and (d) Voting Classifier.

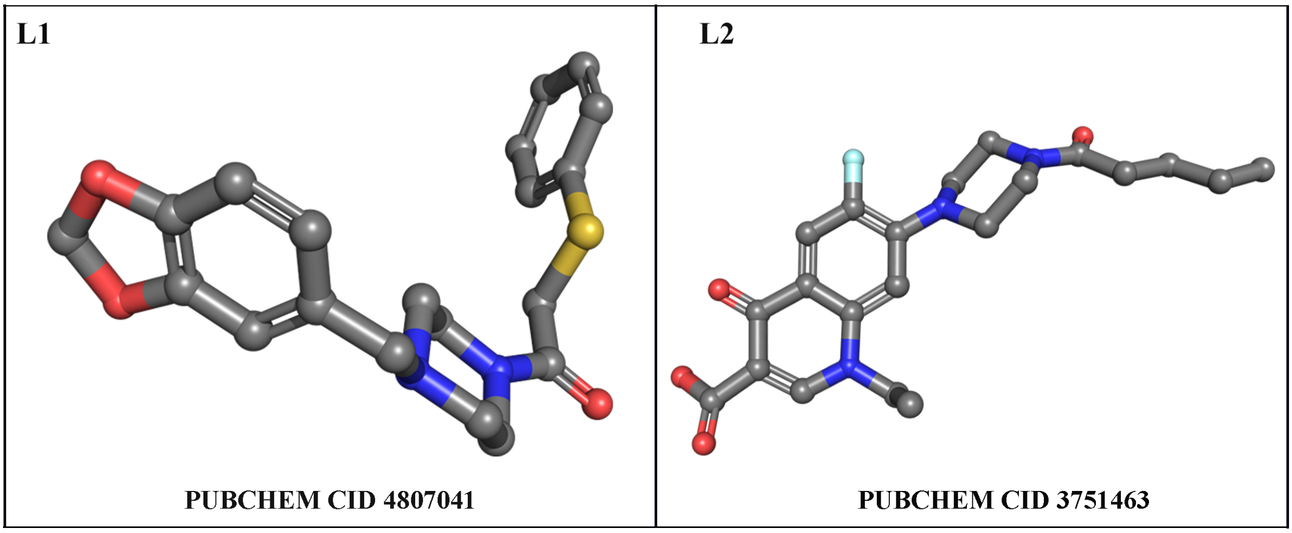

Figure S4: Chemical structures of selected compounds.

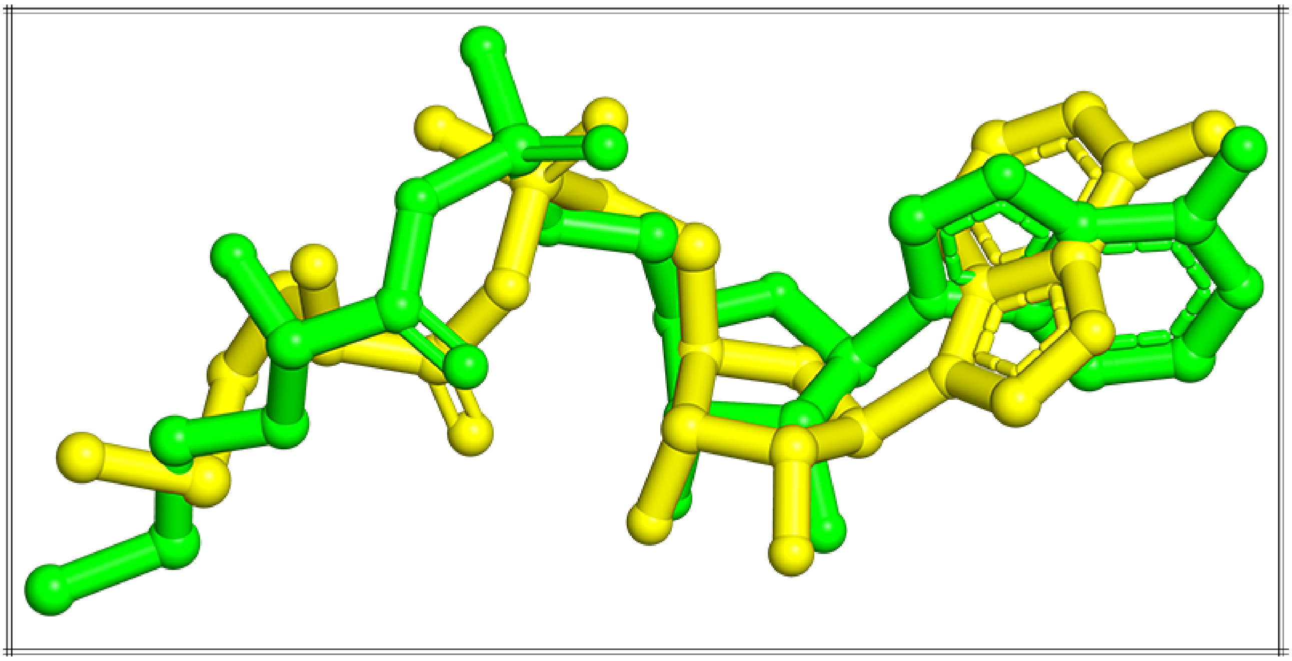

Figure S5: Superimposed of co-crystallized ligand and redocked ligand in the binding site using PyMOL.

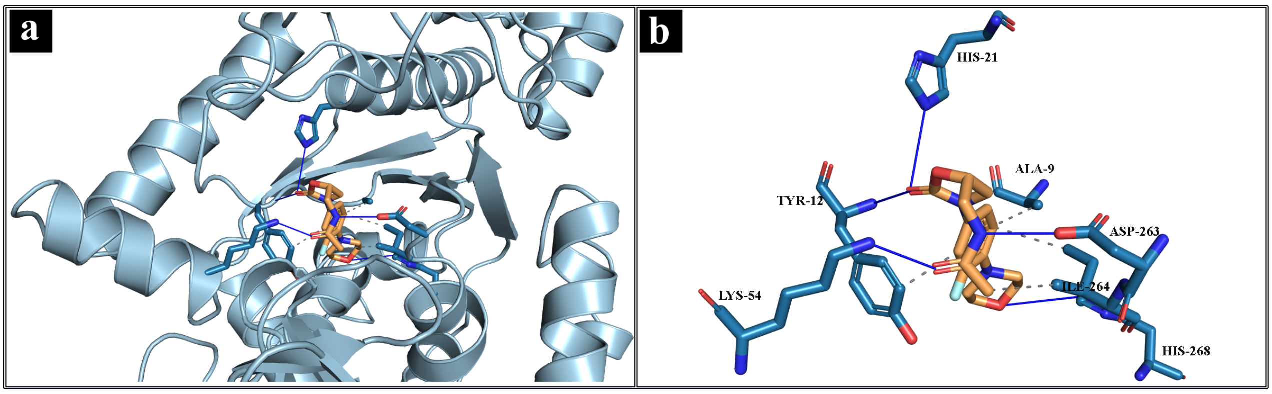

Figure S6: (a) *Mtb*MetRS-Lin complex with ligand in cartoon view, and (b) interaction of active amino acid residues with Lin.

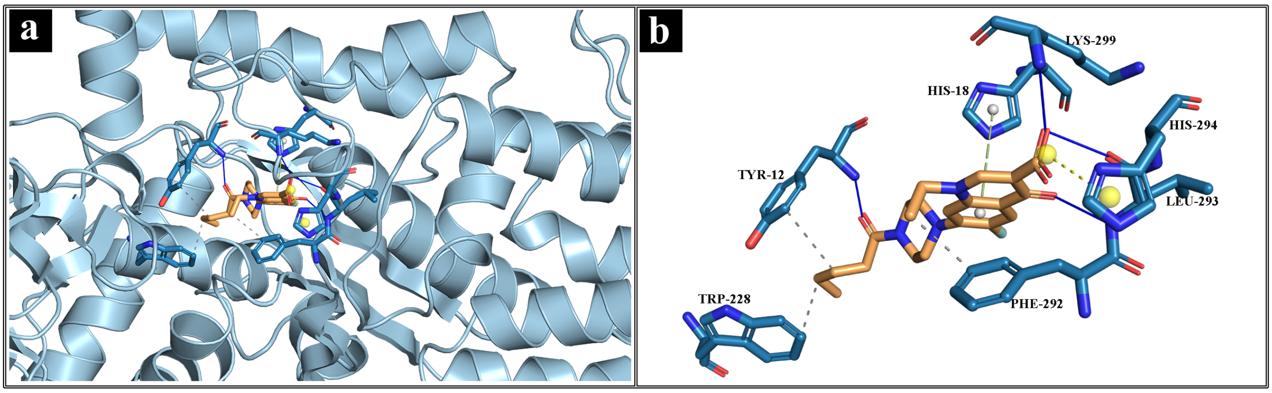

Figure S7: (a) *Mtb*MetRS-L2 complex with ligand in cartoon view, and (b) interaction of active amino acid residues with L2.

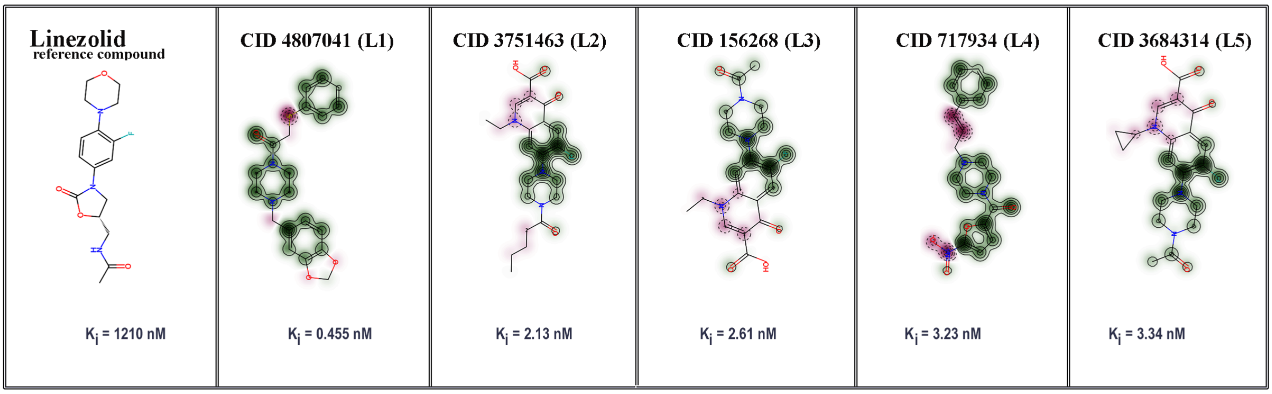

Figure S8: Structural relationships by fingerprint similarity between the reference compound linezolid and the top five molecules (L1 to L5) where K_i is the inhibition constant predicted from molecular docking targeting *Mtb*MetRS. The color codings are green for positive difference, gray for no change in similarity, and pink for negative difference.

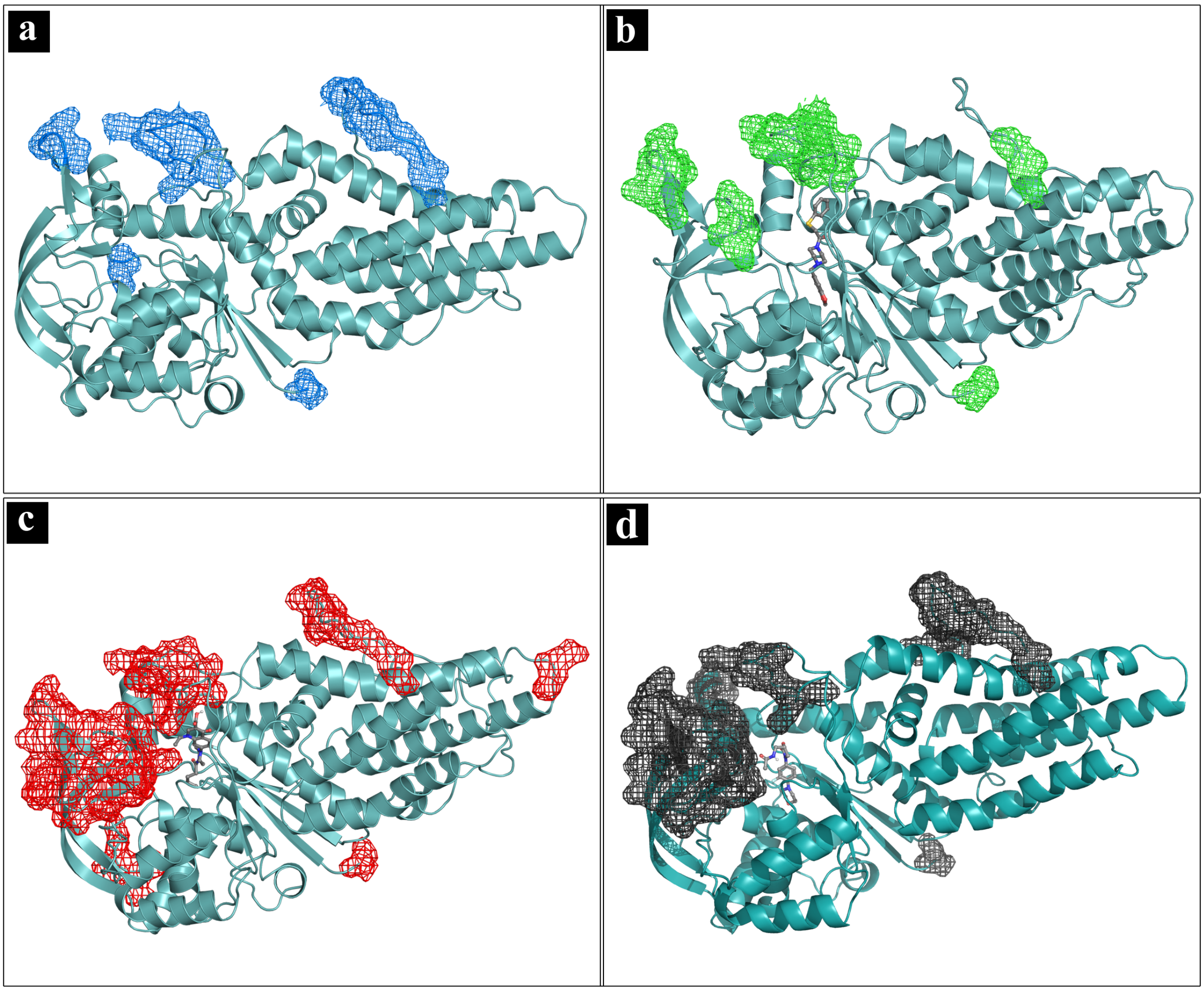

Figure S9: Higher fluctuation region of protein structure in mesh structure with cartoon view (a) Apo, (b) P-L1, (c) P-L2, and (d) P-Lin.

Table S5: Active amino acid residues with RMSF values (Å)

Residues	Apo	P-L1	P-L2	P-Lin
9	0.93	0.73	0.76	0.83
10	0.80	0.61	0.70	0.73
12	0.76	0.77	0.83	1.16
18	0.89	1.08	1.10	0.96
20	0.77	0.95	0.94	0.87
21	0.71	0.87	0.80	0.76
24	0.69	0.71	0.63	0.64
49	1.07	0.79	1.09	0.81
228	0.65	1.21	0.88	1.15
231	0.73	0.89	0.73	0.76
232	0.67	0.95	0.65	0.75
235	0.61	0.58	0.52	0.48
261	0.71	0.68	0.72	0.79
263	0.80	0.82	0.83	1.05
264	0.70	0.69	0.67	0.75
268	0.58	0.54	0.57	0.62
292	0.79	0.96	0.78	0.96
293	0.81	0.92	0.81	0.80

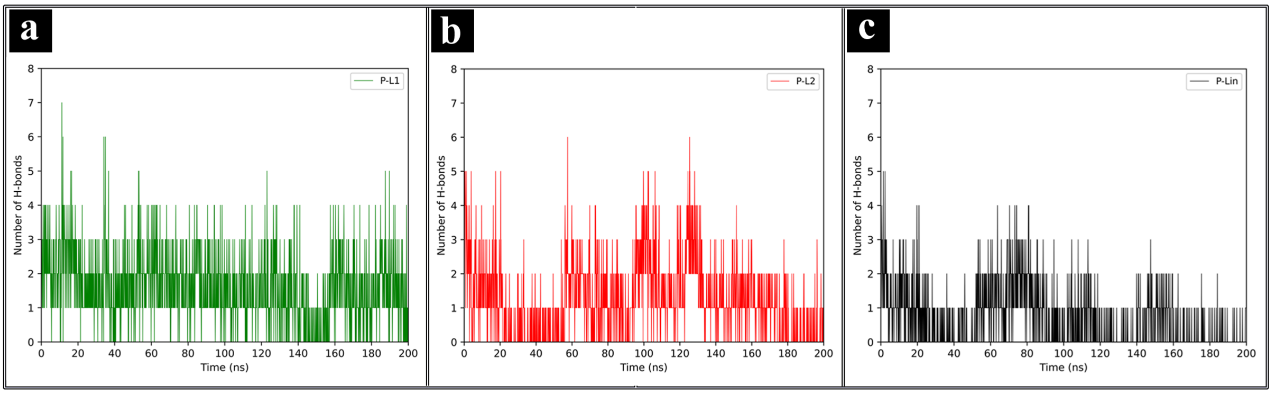

Figure S10: Number of hydrogen bond formed in P-L1 (green), and P-L2 (red) during 200 ns MD simulation.

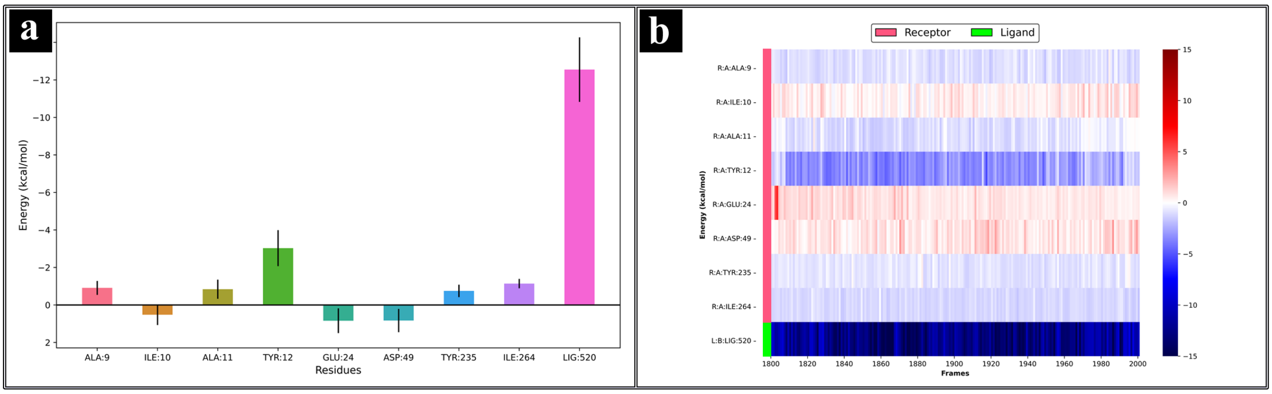

Figure S11: Residues-wise MM/PBSA free energy contribution with cut-off of -0.5 kcal/mol in P-L1 complex during the last 20 ns in form of (a) bar diagram, and (b) heatmap per unit frame.

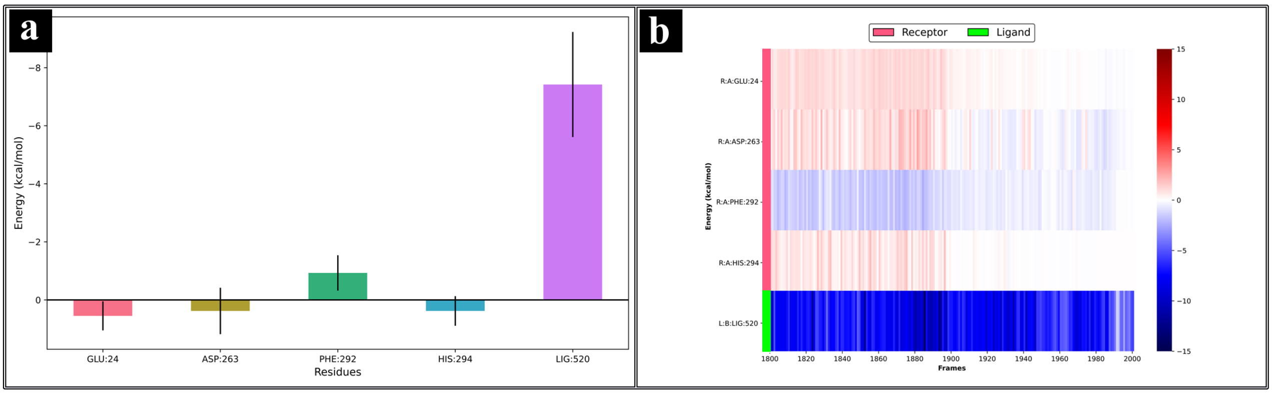

Figure S12: Residues-wise MM/PBSA free energy contribution with cut-off of -0.5 kcal/mol in P-L2 complex during the last 20 ns in form of (a) bar diagram, and (b) heatmap per unit frame.

Table S6: Physicochemical attributes of L1 and L2 by SwissADME server

Compounds	L1	L2
Molecular Weight	370.47	403.45
Octanol/water partition coefficient (logP)	3.08	2.55
Rotatable Bonds	6	7
Number of H-bond acceptors	4	5
Number of H-bond donors	0	1
Bioavailability score	0.55	0.56
Log K_p (cm/s)	-6.37	-6.95

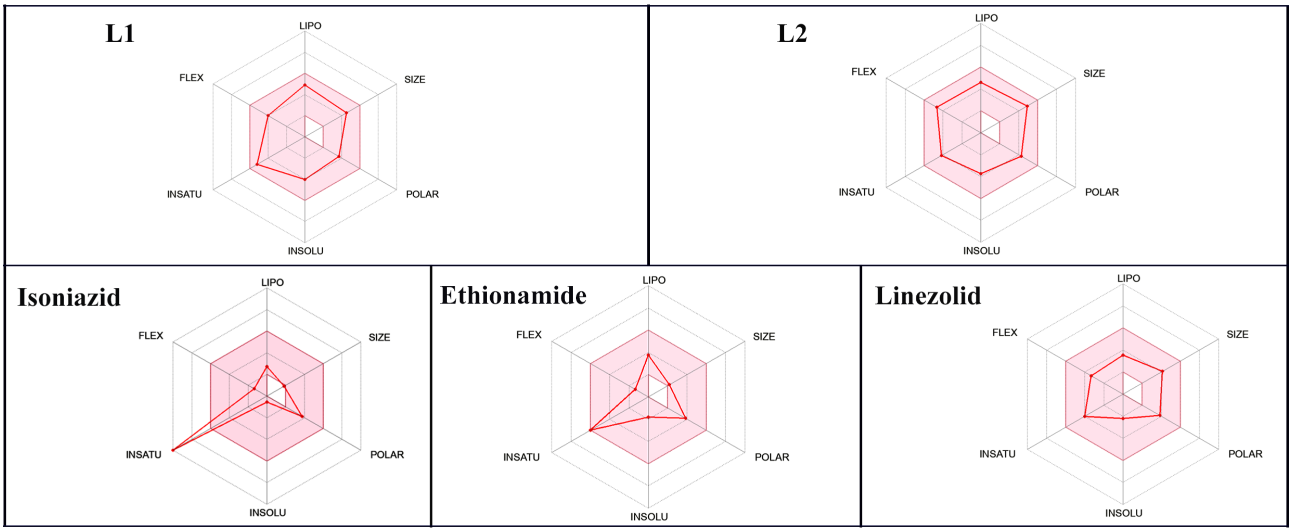

Figure S13: Bioavailability radar diagram for isoniazid, ethionamide, and linezolid as references and compounds L1, and L2.

Table S7: ADMET analysis of L1 and L2 using pkCSM webserver

Property	PARAMETERS	L1	L2
Absorption	Water solubility	-3.792	-3.245
	Caco2 permeability	1.059	1.058
	Intestinal absorption (human)	92.526	94.711
	Skin Permeability	-2.769	-2.735
	P-glycoprotein substrate	Yes	Yes
	P-glycoprotein I inhibitor	Yes	No
	P-glycoprotein II inhibitor	Yes	No
Distribution	VDss (human)	0.727	-1.109
	Fraction unbound (human)	0.082	0.413
	BBB permeability	0.007	-0.379
	CNS permeability	-0.922	-2.637
Metabolism	CYP2D6 substrate	Yes	No
	CYP3A4 substrate	Yes	No
	CYP1A2 inhibitior	No	No
	CYP2C19 inhibitior	Yes	No
	CYP2C9 inhibitior	No	No
	CYP2D6 inhibitior	Yes	No
	CYP3A4 inhibitior	No	No
Excretion	Total Clearance	1.099	0.628
	Renal OCT2 substrate	No	No
Toxicity	AMES toxicity	Yes	No
	Max. tolerated dose (human)	-0.49	0.943
	hERG I inhibitor	No	No
	hERG II inhibitor	Yes	No
	Oral Rat Acute Toxicity (LD50)	2.692	2.646
	Oral Rat Chronic Toxicity (LOAEL)	1.07	1.836
	Hepatotoxicity	Yes	Yes
	Skin Sensitisation	No	No
	T.Pyriformis toxicity	0.899	0.29
	Minnow toxicity	0.476	1.68

Table S8: Toxicity prediction of L1 and L2 using ProTox-II

Particulars	Toxicity prediction (probability) for L1	Toxicity prediction (probability) for L2
Hepatotoxicity	Inactive (0.76)	Inactive (0.65)
Carcinogenicity	Inactive (0.55)	Inactive (0.54)
Immunotoxicity	Active (0.62)	Inactive (0.66)
Mutagenicity	Inactive (0.69)	Active (0.55)
Cytotoxicity	Inactive (0.61)	Inactive (0.65)

Table S9: LD_{50} value of L1 and L2

Particulars	L1	L2
Predicted LD_{50}	750 mg/kg	4000 mg/kg

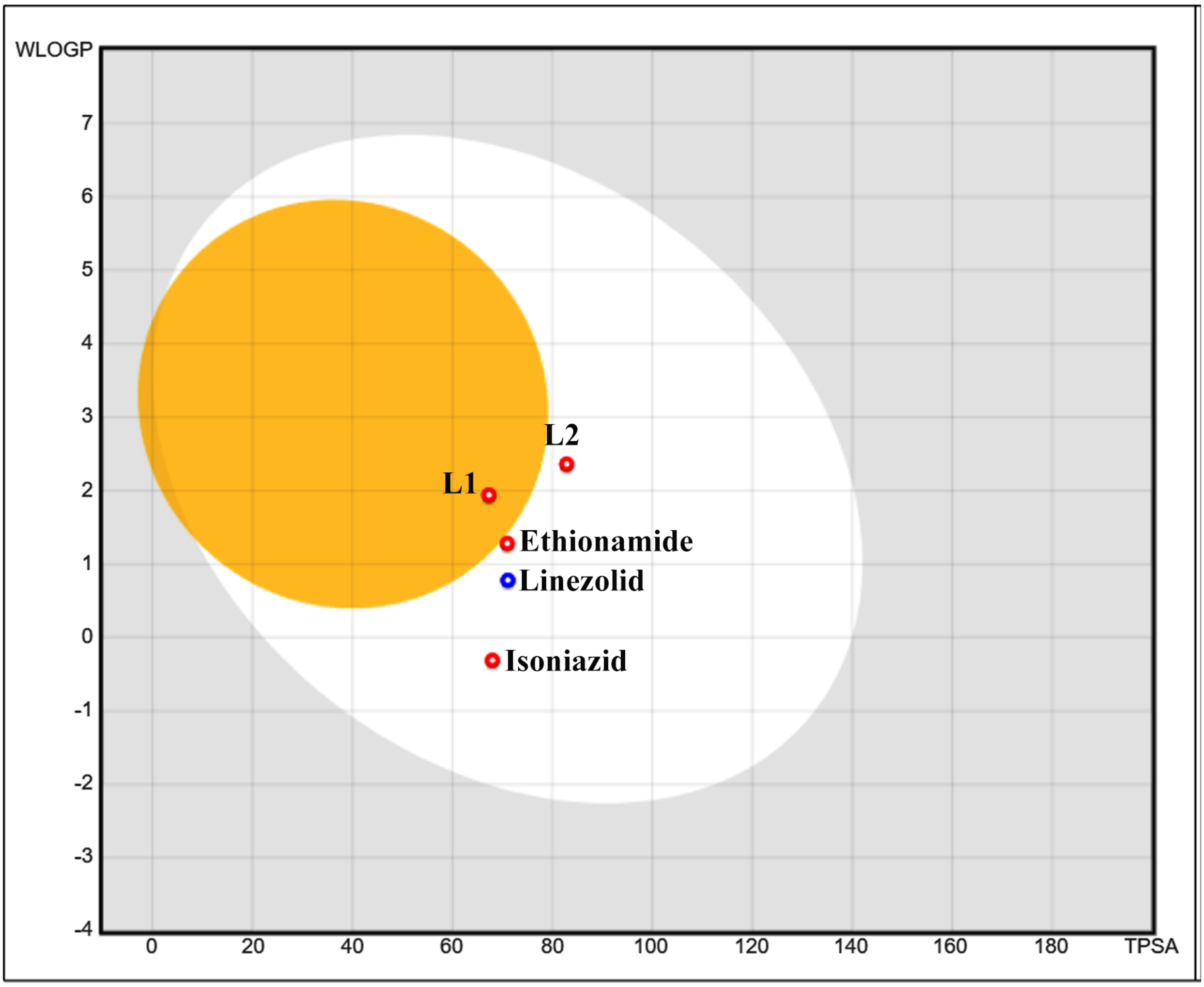

Figure S14: Boiled-Egg graph of isoniazid, ethionamide, and linezolid as references and compounds L1, and L2.
